# Controllable Thrombolysis Using a Nanobubble-Imaging-Guided rtPA Targeted Delivery Strategy

**DOI:** 10.34133/bmef.0040

**Published:** 2024-03-26

**Authors:** Jian Tang, Huiting Xu, Mingxi Li, Yang Liu, Fang Yang

**Affiliations:** State Key Laboratory of Digital Medical Engineering, Jiangsu Key Laboratory for Biomaterials and Devices, School of Biological Sciences and Medical Engineering, Southeast University, Nanjing 210096, China.

## Abstract

**Objective:** The objective of this work is to design and fabricate a novel multifunctional nanocarrier combining thrombus-targeted imaging and ultrasound-mediated drug delivery for the theranostics of thrombotic diseases. **Impact Statement:** This study develops a new technology that can accurately visualize the thrombus and deliver drugs with controllable properties to diagnose and treat thrombotic diseases. **Introduction:** Thrombotic diseases are a serious threat to human life and health. The diagnosis and treatment of thrombotic diseases have always been a challenge. In recent years, nanomedicine has brought new ideas and new methods for the theranostics of thrombotic diseases. However, there are also many problems need to be solved, such as biosafety and stability of nanocarriers, early diagnosis, and timely treatment of thrombotic diseases, difficulty in clinical translation. **Methods:** The S1P@CD-PLGA-rtPA nanobubbles (NBs) were prepared by integrating sulfur hexafluoride (SF_6_)-loaded poly (D, L-lactide-co-glycolide) (PLGA) NBs, cyclodextrin (CD), sphingosine-1-phosphate (S1P), and recombinant tissue plasminogen activator (rtPA). **Results:** S1P@CD-PLGA-rtPA NBs had rapid and excellent thrombosis targeting imaging performance based on the specific interaction of S1P–S1PR1 (sphingosine-1-phosphate receptor 1). Furthermore, S1P@CD-PLGA-rtPA NBs that specifically targeting to the thrombosis regions could also respond to external ultrasound to achieve accurate and efficient delivery of rtPA to enhance the thrombolysis effectiveness and efficiency. **Conclusion:** This study proposes a new idea and strategy of targeting thrombus in rats via the specific interaction of S1P–S1PR1. On this basis, the acoustic response properties of bubble carriers could be fully utilized by combining thrombus-specific targeted imaging and ultrasound-mediated drug delivery for effective thrombolysis, which is expected to be applied in targeted diagnosis and treatment of thrombotic diseases in the future.

## Introduction

Thrombotic diseases, such as acute myocardial infarction [1], pulmonary embolism [2], deep venous thrombosis [3], and ischemic stroke [4], are the leading cause of most morbidity and mortality worldwide [5]. Vascular embolism caused by thrombus can lead to severe tissue damage [6] and organ failure [7], which is ultimately life-threatening. Thus, it is of great importance for the early diagnosis and timely treatment of thrombotic diseases. Nowadays, drug administration [8], percutaneous intervention [9] and surgical thrombectomy [10] are 3 main clinical treatments for thrombotic diseases. However, the current thrombolysis strategies offer limited results due to the short half-lives of therapeutic drugs, low targeting ability and unexpected bleeding complications.

In recent years, nanomedicine has brought new strategies and new methods for the diagnosis and treatment of thrombotic diseases [11], such as a cyclic arginine-glycine-aspartic acid (cRGD) peptide modified thrombus-targeted nanobubbles (NBs) [12], click chemically mediated thrombus-targeted microbubbles [13], fibrin site-specific nanoprobe for thrombus imaging [14], engineered nanoplatelets for theranostic of thrombus [15], magnetically guided thrombus-targeted microbubble platform [16], platelet-derived porous nanomotor for thrombus therapy [17], and so on, which has achieved dramatic results in the theranostic of thrombus in vitro and in vivo. In particular, there has been a great deal of interest in the strategy of sonothrombolysis with targeted nano/microbubbles. The promoting effect of ultrasound combined with drug-loaded nano/microbubbles on thrombolysis has been confirmed in numerous in vitro and in vivo reports [18–21]. Furthermore, the intravascular sonothrombolysis with different micro/nanocarriers has also exhibited great potential in the field of sonothrombolysis applications, such as a mixture of NBs and microbubbles [22], combination of magnetic microbubbles and nanodroplets [23], and combination of nanodroplets and tissue plasminogen activator (tPA) [24].

Nevertheless, there are still some problems needed to be solved [25]: (a) the biosafety and stability of nanocarriers in vivo; (b) the early and accurate diagnosis and treatment of thrombotic diseases; and (c) the complicated construction system and the difficulty of clinical translation.

Therefore, to overcome the problems related to the current nanomedicine in theranostic of thrombosis, we designed and prepared S1P@CD-PLGA-rtPA NBs by integrating sulfur hexafluoride (SF_6_)-loaded poly (D, L-lactide-co-glycolide) (PLGA) NBs with excellent ultrasound imaging enhancement effect, cyclodextrin (CD) with unique encapsulation properties, sphingosine-1-phosphate (S1P) with thrombosis targeting effect and clinical first-line thrombolytic drug recombinant tissue plasminogen activator (rtPA). The characterization results showed that S1P@CD-PLGA-rtPA NBs diameters of approximately 220 nm exhibited stable properties, good biosafety, and excellent ultrasound imaging enhancement effect. The results of in vitro drug release experiments and in vitro thrombolysis experiments demonstrated that the controlled release of rtPA has been realized by exposure to the external ultrasound due to the ultrasonic responsive property of S1P@CD-PLGA-rtPA NBs. Furthermore, in both rat mesenteric arterioles thrombosis model and rat inferior vena cava thrombosis model, it was confirmed that S1P@CD-PLGA-rtPA NBs rapidly target to the thrombosis due to S1P–S1PR1 (sphingosine-1-phosphate receptor 1) axis in thrombosis regions to be imaged to delineate the thrombus lesions. The excellent thrombolysis effectiveness and efficiency was realized when ultrasound was applied to the S1P@CD-PLGA-rtPA NBs that targeted thrombus lesions due to controlled release of rtPA. Our results indicate that this technology can accurately visualize the thrombus and deliver drugs with controllable properties to diagnose and treat the thrombotic diseases.

## Results and Discussion

### Characterization of S1P@CD-PLGA-rtPA NBs

**Fig. 1.**
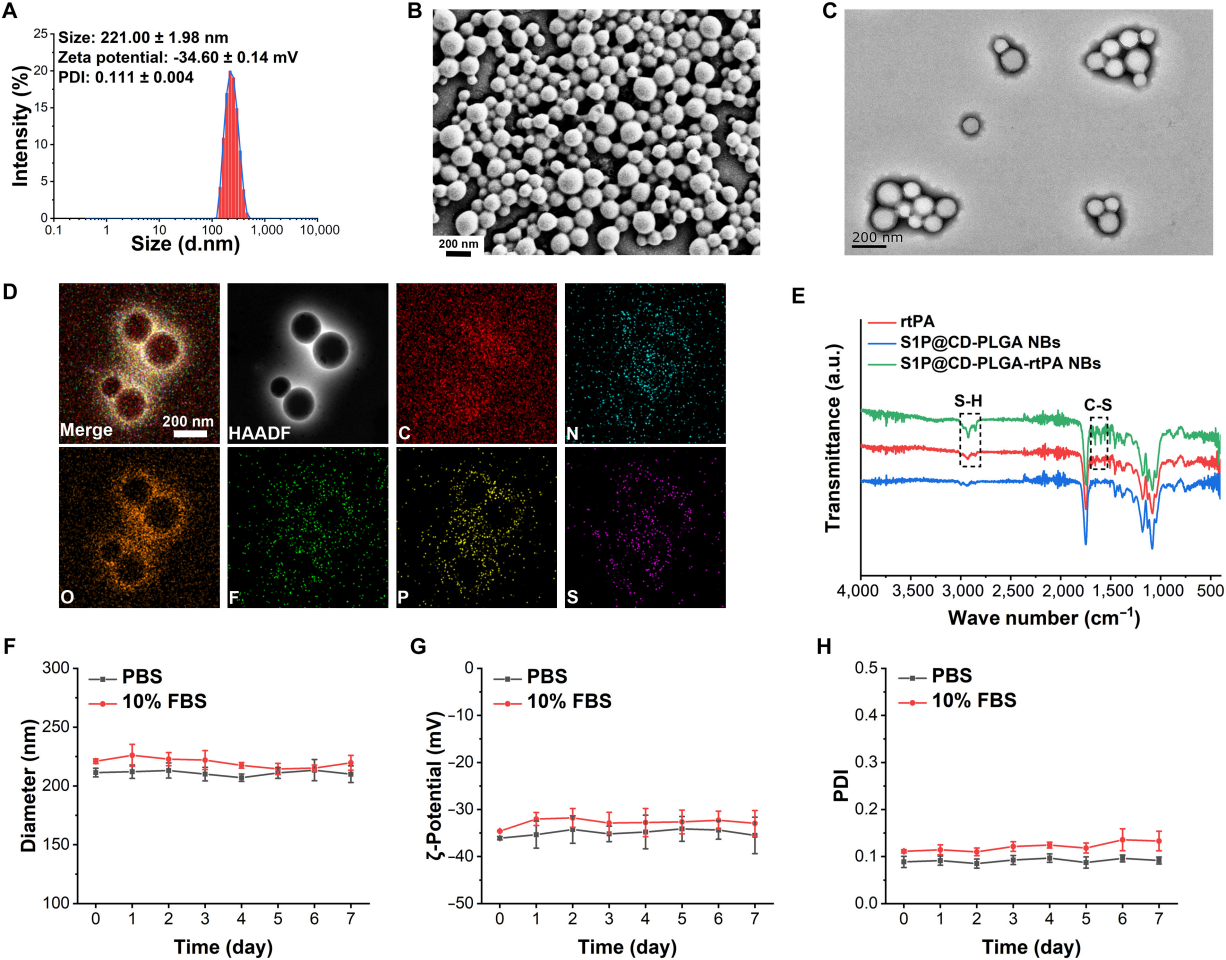
Characterization of S1P@CD-PLGA-rtPA NBs. (A) Hydrodynamic size, zeta potential, PDI, and size distribution of S1P@CD-PLGA-rtPA NBs. (B) SEM morphological image, (C) TEM morphological image, and (D) distribution mapping of element composition of S1P@CD-PLGA-rtPA NBs. (E) Infrared absorption spectra of rtPA, S1P@CD-PLGA NBs, and S1P@CD-PLGA-rtPA NBs. (F) The in vitro stability of size, (G) zeta potential, and (H) PDI of S1P@CD-PLGA-rtPA NBs stored in PBS (pH = 7.4) and 10% FBS for 7 d. Error bars: mean ± SD (*n* = 3).

The dynamic light scattering results showed that the hydrodynamic size of S1P@CD-PLGA-rtPA NBs was 221.00 ± 1.98 nm with polydispersity index (PDI) of 0.111 ± 0.004, and the zeta potential was −34.60 ± 0.14 mV (Fig. 1A). The scanning electron microscope (SEM) morphological image indicated that the S1P@CD-PLGA-rtPA NBs appeared as well-dispersed spherical structures (Fig. 1B). The transmission electron microscope (TEM) morphological image in Fig. 1C showed that the S1P@CD-PLGA-rtPA NBs had a round appearance and unifom size with the structure of clear PLGA shell and hollow gas core. Moreover, the distribution mapping of element composition indicated the S1P@CD-PLGA-rtPA NBs are mainly composed of carbon (C), nitrogen (N), oxygen (O), fluorine (F), phosphorus (P) and sulfur (S) (Fig. 1D). Among them, C and O are the main component elements of PLGA shell, F and S are the constituent elements of SF_6_, and N and P on the surface of PLGA shell are the unique elements of amino-β-CD and S1P, respectively. S inside the PLGA shell is the unique element of rtPA, indicating the successful assembly of S1P@CD-PLGA-rtPA NBs. The elemental contents of carbon (C), nitrogen (N), oxygen (O), fluorine (F), phosphorus (P), and sulfur (S) elements in S1P@CD-PLGA-rtPA NBs were displayed in Fig. S1.

The infrared absorption spectra of rtPA, S1P@CD-PLGA NBs and S1P@CD-PLGA-rtPA NBs in Fig. 1E showed that, compared with the absorption peak of the infrared absorption spectrum of S1P@CD-PLGA NBs, the infrared absorption spectrum of S1P@CD-PLGA-rtPA NBs added a unique S–H vibrational absorption peak (668 cm^−1^) (Fig. S2) and C–S vibrational absorption peak (1,560 cm^−1^) (Fig. S3) of rtPA, demonstrating the successful loading of rtPA in S1P@CD-PLGA-rtPA NBs.

In addition, the in vitro stability of S1P@CD-PLGA-rtPA NBs stored in phosphate-buffered saline (PBS) (pH = 7.4) and 10% fetal bovine serum (FBS) for 7 d at 25 °C was characterized and the results showed that the S1P@CD-PLGA-rtPA NBs had no marked change of particle size (Fig. 1F), zeta potential (Fig. 1G), and PDI (Fig. 1H), which indicated the S1P@CD-PLGA-rtPA NBs had a good in vitro stability. Furthermore, the encapsulation efficiency and rtPA loading content in S1P@CD-PLGA-rtPA NBs was 77.03 ± 0.27% and 0.42 ± 0.07%, respectively.

### In vitro ultrasound imaging enhancement of S1P@CD-PLGA-rtPA NBs

**Fig. 2.**
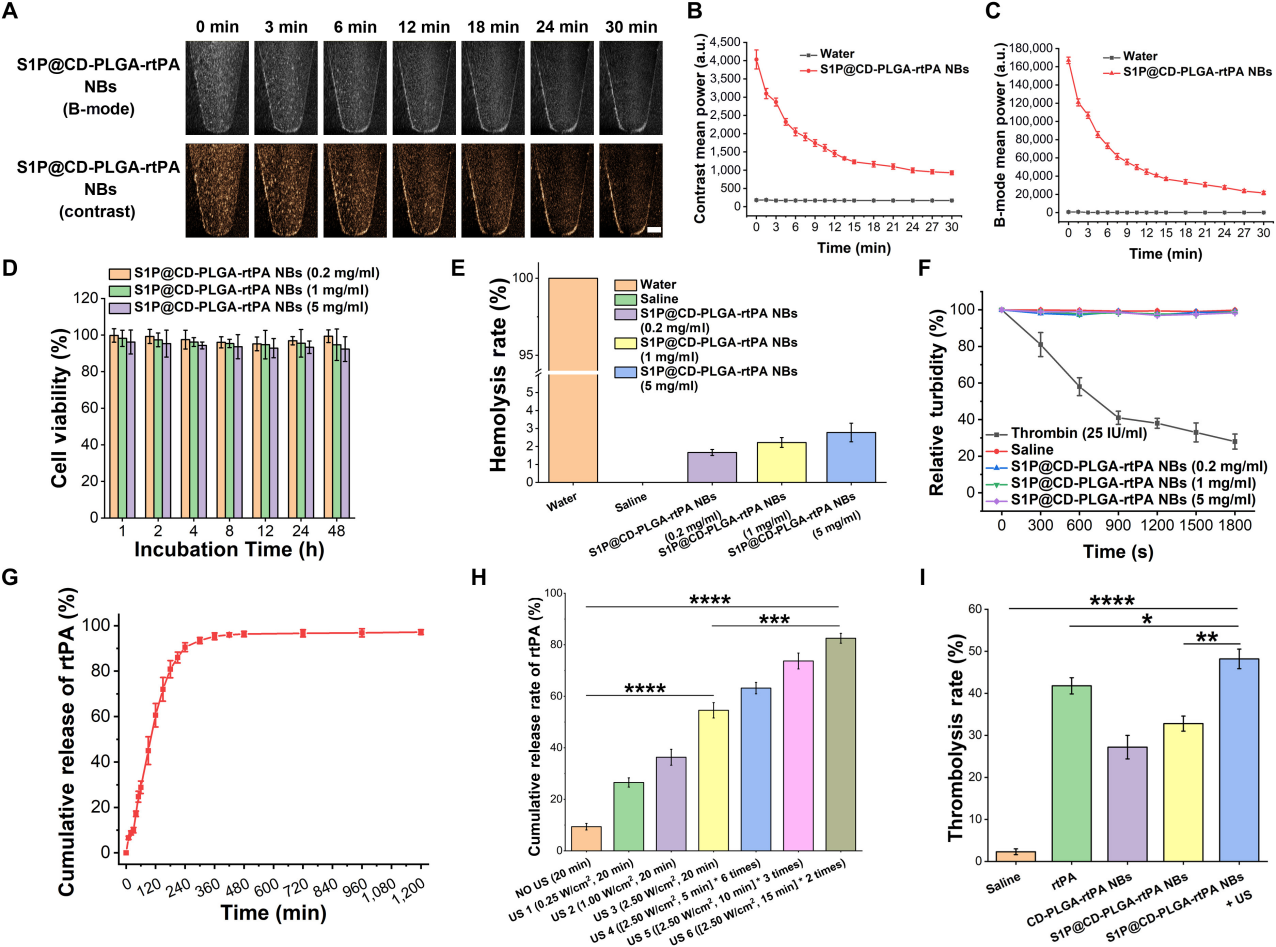
Characterization of in vitro ultrasound imaging enhancement, biosafety, in vitro release of rtPA, and in vitro thrombolysis of S1P@CD-PLGA-rtPA NBs. (A) B-mode and contrast mode ultrasound images of S1P@CD-PLGA-rtPA NBs at different time intervals (scale bar: 2 mm). Quantitative analysis of the mean power in (B) contrast mode and (C) B-mode of water and S1P@CD-PLGA-rtPA NBs. (D) Cell viability of different concentrations of S1P@CD-PLGA-rtPA NBs (0.2, 1, and 5 mg/ml) incubated with rat carotid endothelial cells for different times (1, 2, 4, 8, 12, 24, and 48 h). (E) Hemolysis rate of water, saline, and different concentrations of S1P@CD-PLGA-rtPA NBs (0.2, 1, and 5 mg/ml) incubated with rat erythrocytes for 4 h. (F) Platelet aggregation assay in which rat platelets were mixed with thrombin, saline, and different concentrations of S1P@CD-PLGA-rtPA NBs (0.2, 1, and 5 mg/ml). (G) In vitro release profiles of rtPA in S1P@CD-PLGA-rtPA NBs and (H) in vitro release of rtPA under different ultrasonic strategies. (I) In vitro thrombolysis experiments with the addition of saline, rtPA, CD-PLGA-rtPA NBs, S1P@CD-PLGA-rtPA NBs, and S1P@CD-PLGA-rtPA NBs + US, respectively (2 h). Error bars: mean ± SD (*n* = 3). The statistical significance is indicated by ^*^ *P* < 0.05, ^**^ *P* < 0.01, ^***^ *P* < 0.001, and ^****^ *P* < 0.0001, determined using one-way ANOVA.

The performance of NBs ultrasound imaging enhancement greatly affects the realization of subsequent thrombosis-targeted ultrasound imaging. Therefore, the in vitro ultrasound imaging effect of S1P@CD-PLGA-rtPA NBs within 30 min was characterized, and the results in Fig. 2A and Fig. S4 confirmed that, compared to water, S1P@CD-PLGA-rtPA NBs had an excellent ultrasound-enhanced imaging effect owing to the fact that SF_6_ gas-core structure of NBs greatly enhance the reflection and scattering of ultrasonic waves. Besides, the quantitative results of contrast mean power (Fig. 2B) and B-mode mean power (Fig. 2C) of in vitro ultrasound images within 30 min demonstrated that S1P@CD-PLGA-rtPA NBs had excellent ultrasound imaging enhancement effect compared with water.

### Biosafety evaluation of S1P@CD-PLGA-rtPA NBs

First, the toxicity of S1P@CD-PLGA-rtPA NBs at different concentrations on rat carotid endothelial cells was studied. The results in Fig. 2D showed that incubation of endothelial cells with S1P@CD-PLGA-rtPA NBs at different concentrations (0.2, 1, and 5 mg/ml) for different time (1, 2, 4, 8, 12, 24, and 48 h) had no adverse effect on cell viability, which confirmed that S1P@CD-PLGA-rtPA NBs were biocompatible and noncytotoxic.

Second, the hemolysis effect of S1P@CD-PLGA-rtPA NBs at different concentrations (0.2, 1, and 5 mg/ml) on erythrocytes was studied to test the blood compatibility of the NBs. As shown in Fig. 2E, the results of erythrocyte hemolysis assay showed that the hemolysis rate of S1P@CD-PLGA-rtPA NBs was 1.67 ± 0.17% (NBs at a concentration of 0.2 mg/ml), 2.22 ± 0.27% (NBs at a concentration of 1 mg/ml), and 2.78 ± 0.52% (NBs at a concentration of 5 mg/ml). Therefore, the hemolysis rates of S1P@CD-PLGA-rtPA NBs at different concentrations (0.2, 1, and 5 mg/ml) were all less than 5% [26]. Thus, it could be concluded that S1P@CD-PLGA-rtPA NBs had no obvious hemolysis effect on erythrocytes.

Third, the platelet aggregation assay was performed to verify the effect of S1P@CD-PLGA-rtPA NBs on platelet aggregation function [27]. The results of platelet aggregation assays indicated that thrombin rapidly induced platelet aggregation, while saline and S1P@CD-PLGA-rtPA NBs did not cause platelet aggregation, resulting in the reduced risk of secondary thrombosis (Fig. 2F). In summary, the prepared S1P@CD-PLGA-rtPA NBs had good hemocompatibility for the subsequent in vivo application of thrombolysis.

### In vitro rtPA release property of S1P@CD-PLGA-rtPA NBs

As shown in Fig. 2G, the in vitro release curve of rtPA in S1P@CD-PLGA-rtPA NBs indicated that the release of rtPA was smooth without sudden release and the cumulative drug release rate was 28.88 ± 2.72% at 1 h, 60.61 ± 5.18% at 2 h, and 90.57% ± 1.94% at 4 h, which basically reached the plateau of drug release. Moreover, based on the acoustic response properties of the NB carriers, the in vitro release of rtPA in S1P@CD-PLGA-rtPA NBs under different external ultrasonic strategies was further studied. The results in Fig. 2H revealed that the application of external ultrasound could effectively promote the rapid and efficient release of rtPA from NBs. Specifically, the cumulative release rate of rtPA was 9.37 ± 1.27% (NO US), 26.53 ± 1.79% (US 1), 36.33 ± 3.09% (US 2), 54.57 ± 2.98% (US 3), 63.17 ± 2.22% (US 4), 73.70 ± 3.08% (US 5), and 82.53 ± 1.91% (US 6). It could be found that the increase of the ultrasonic intensity was beneficial to accelerate the in vitro release of rtPA from NBs. For the safety consideration, the thermal effect with ultrasound excitations is an issue that deserves attention [28,29]. The method of multiple interval ultrasound could enhance the release of rtPA and reduce the thermal effect caused by ultrasound excitations compared with the method of single continuous ultrasound. Therefore, the external ultrasonic strategy (US 6: frequency of 1 MHz, intensity of 2.50 W/cm^2^, ultrasonic for 15 min, pause for 5 min, and then ultrasonic for another 15 min) was applied for subsequent experiments.

### In vitro thrombolysis effect of S1P@CD-PLGA-rtPA NBs

In vitro thrombolysis experiments were performed to evaluate the thrombolysis effect of different treating groups (saline was used as a negative control group and the content of rtPA remained the same in other groups). The results in Fig. 2I showed the thrombolysis rate was 2.31 ± 0.57% (Saline), 41.85 ± 1.93% (rtPA), 27.22 ± 2.82% (CD-PLGA-rtPA NBs), 32.83 ± 1.80% (S1P@CD-PLGA-rtPA NBs), and 48.27 ± 2.33% (S1P@CD-PLGA-rtPA NBs + US), respectively. The S1P@CD-PLGA-rtPA NBs did not show much improvement over the rtPA alone, and the reason may be that free rtPA could directly contact the thrombus clots and exert thrombolytic effects, and there was no metabolism of rtPA. However, as for S1P@CD-PLGA-rtPA NBs, the encapsulated rtPA could only exert thrombolytic effects after releasing naturally, and the release rate of rtPA was 60.61 ± 5.18% at 2 h (Fig. 2G). Therefore, the content of rtPA released by the S1P@CD-PLGA-rtPA NBs group was lower than that of free rtPA at the same time. Crucially, it was confirmed that the thrombolysis strategy of S1P@CD-PLGA-rtPA NBs combined with the external ultrasound had the optimal in vitro thrombolysis effect. The reason may be that: (a) The activity of rtPA in S1P@CD-PLGA-rtPA NBs was better preserved compare with free rtPA. (b) The rtPA in the S1P@CD-PLGA-rtPA NBs was fully released under the external ultrasound. (c) Ultrasonic cavitation enhanced the thrombus penetration depth of rtPA, which benefits the dissolution inside the thrombus clots.

### In vivo thrombosis targeting imaging of S1P@CD-PLGA-rtPA NBs in rat models

The mesenteric arterioles thrombosis model [30] and inferior vena cava thrombosis model [31] induced by FeCl_3_ are important tools for the study of arterial and venous thrombus, respectively. Thus, rat models of ferric chloride-induced mesenteric arterioles thrombosis and inferior vena cava thrombosis were constructed to explore the arterial and venous thrombosis targeting properties of S1P@CD-PLGA-rtPA NBs.

**Fig. 3.**
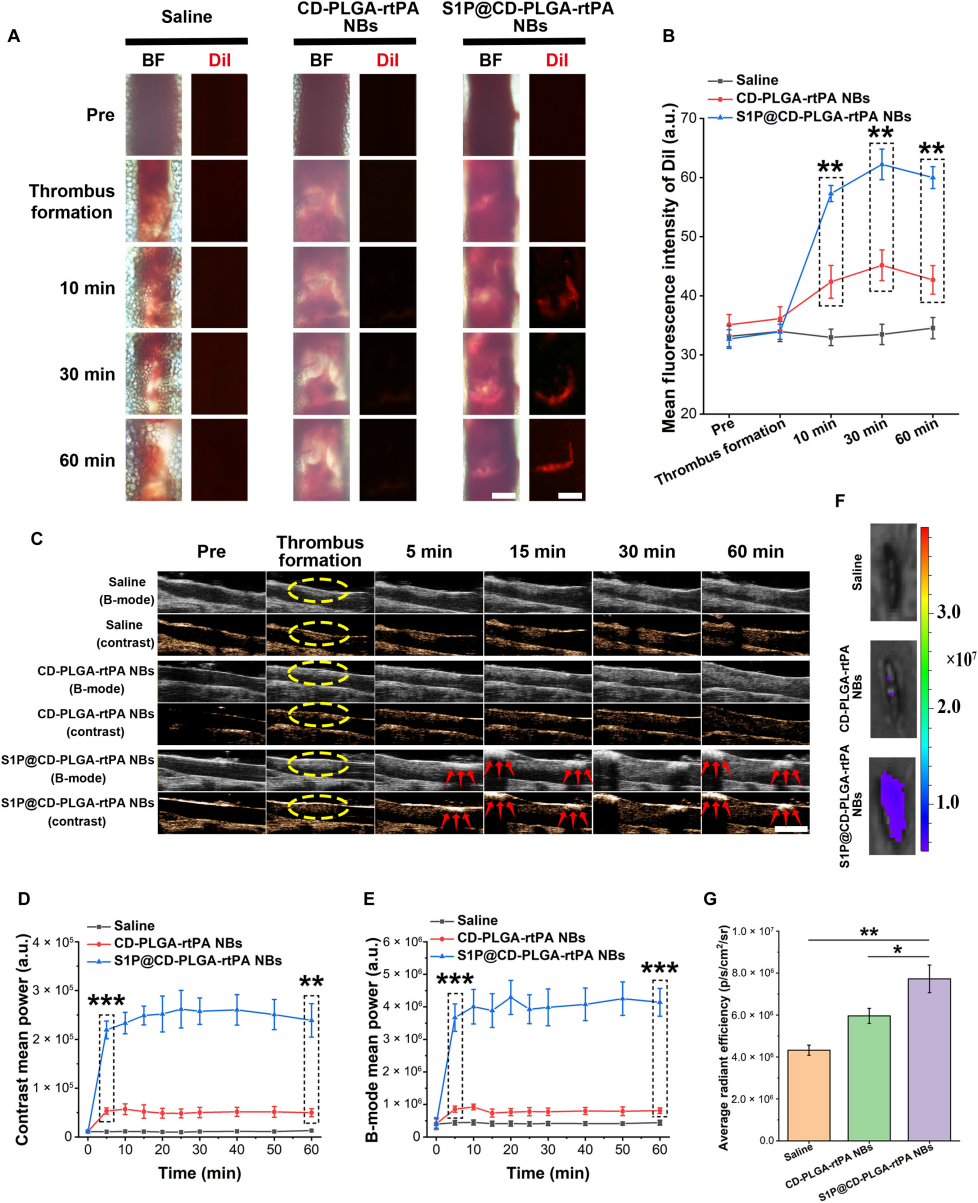
In vivo thrombosis targeting imaging of S1P@CD-PLGA-rtPA NBs in rat models. (A) Targeted fluorescence imaging of DiI-labeled S1P@CD-PLGA-rtPA NBs in rat mesenteric arterioles thrombosis model (scale bar: 200 μm) and (B) quantitative fluorescence results of intravascular DiI. (C) B-mode and contrast mode ultrasound images of saline, CD-PLGA-rtPA NBs, and S1P@CD-PLGA-rtPA NBs in rat inferior vena cava thrombosis model at different time intervals, respectively. The yellow frame represents the area of thrombus, and red arrows point to the adhered S1P@CD-PLGA-rtPA NBs (scale bar: 2 mm). (D) Quantitative of contrast mean power and (E) B-mode mean power of ultrasound images. (F) Ex vivo near-infrared fluorescence imaging of excised inferior vena cava after intravenous injection of saline, DiR-labeled CD-PLGA-rtPA NBs, and DiR-labeled S1P@CD-PLGA-rtPA NBs for 60 min, respectively. (G) Fluorescence quantitative results of near-infrared fluorescence images. Error bars: mean ± SD (*n* = 3). The statistical significance is indicated by ^*^ *P* < 0.05, ^**^ *P* < 0.01, ^***^ *P* < 0.001, determined using one-way ANOVA.

First, rat models of mesenteric arterioles thrombosis was performed to explore the arterial thrombosis targeting properties of S1P@CD-PLGA-rtPA NBs. As shown in Fig. 3A, the optical microscopy results showed that white clots appeared in the blood vessels when thrombosis formation. After caudal intravenous injection of 1,1'-dioctadecyl-3,3,3',3'-tetramethylindocarbocyanine perchlorate (DiI)-labeled S1P@CD-PLGA-rtPA NBs for 10 min, it was observed that the S1P@CD-PLGA-rtPA NBs rapidly targeted thrombosis regions and remarkbly enhanced the red fluorescence intensity of the thrombosis regions, while there was no obvious red fluorescence in the thrombosis regions by injection of saline and CD-PLGA-rtPA NBs. Moreover, stable targeting of DiI-labeled S1P@CD-PLGA-rtPA NBs was still observed at the thrombosis regions after intravenous injection for 30 and 60 min. Besides, the mean fluorescence intensity of intravascular DiI by injection of S1P@CD-PLGA-rtPA NBs were much higher than that by injection of saline and CD-PLGA-rtPA NBs at the same observation time (Fig. 3B). Thus, it proved that S1P@CD-PLGA-rtPA NBs could rapidly and stably target the thrombosis regions of the rat mesenteric arterioles.

In addition, rat models of inferior vena cava thrombosis was performed to explore the venous thrombosis targeting properties of S1P@CD-PLGA-rtPA NBs. As shown in Fig. 3C, the yellow frame indicated the thrombus formation. The S1P@CD-PLGA-rtPA NBs could rapidly target thrombosis regions after 5 min of caudal intravenous injection as the red arrows indicated and maintain excellent contrast-enhanced ultrasound imaging effect of the thrombosis regions within 60 min. Moreover, the contrast mean power (Fig. 3D) and B-mode mean power (Fig. 3E) of the thrombosis regions by injection of S1P@CD-PLGA-rtPA NBs were much higher than that by injection of saline and CD-PLGA-rtPA NBs at the same observation time. Furthermore, the ex vivo near-infrared fluorescence images and fluorescence quantitative results of excised inferior vena cava after intravenous injection of saline, 1, 1'-dioctadecyl-3,3,3',3'-tetramethylindotricarbocyanine iodide (DiR)-labeled CD-PLGA-rtPA NBs, and DiR-labeled S1P@CD-PLGA-rtPA NBs for 60 min were displayed in Fig. 3F and G. The excised inferior vena cava by injection of S1P@CD-PLGA-rtPA NBs showed the strongest fluorescence ([7.72 ± 0.66] × 10^6^) compared with injection of saline ([4.32 ± 0.24] × 10^6^) and CD-PLGA-rtPA NBs ([5.96 ± 0.36] × 10^6^). Thus, it demonstrated the excellent in vivo targeting effect of S1P@CD-PLGA-rtPA NBs on rat inferior vena cava thrombosis.

**Fig. 4.**
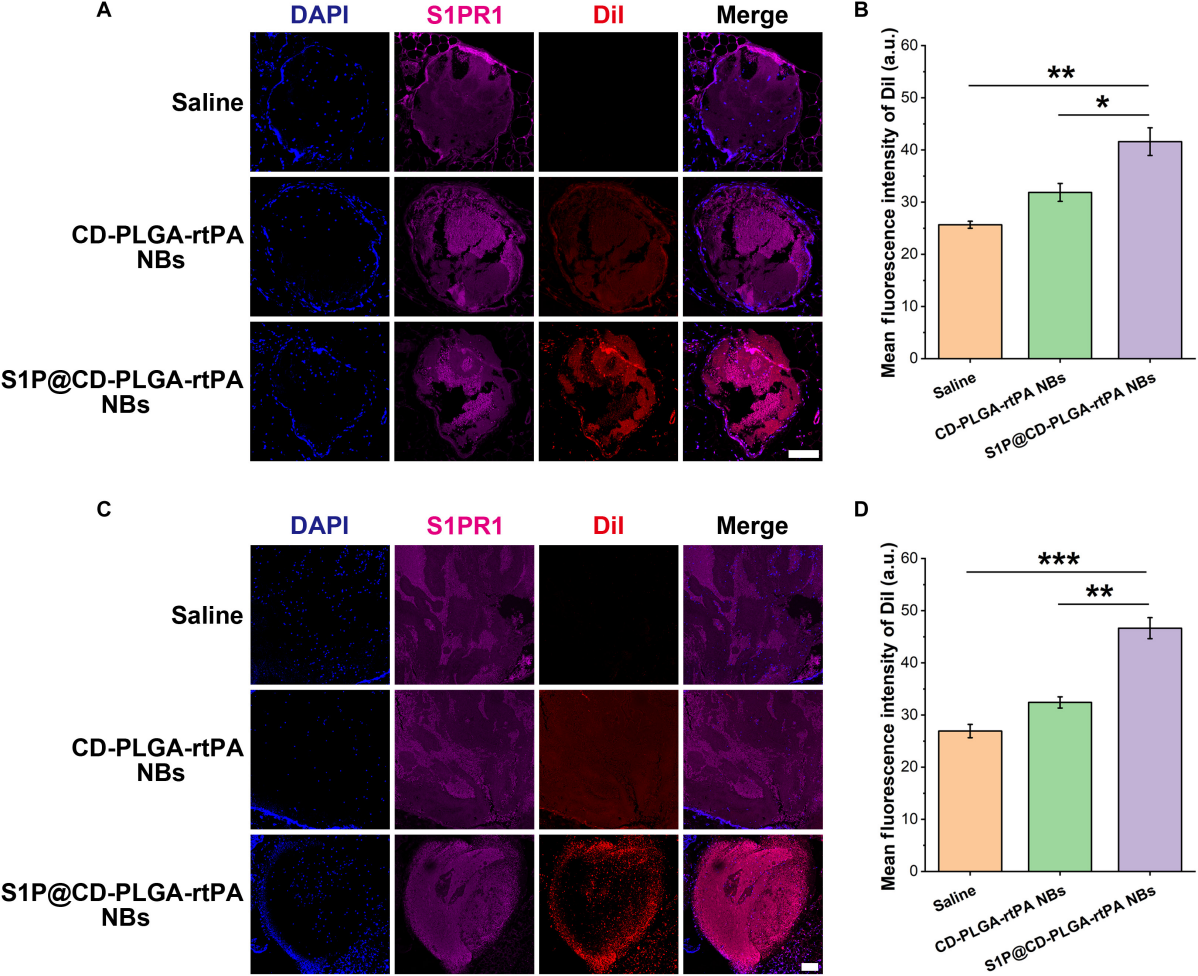
S1PR1 immunofluorescence staining of rat thrombus sections and colocalization with S1P@CD-PLGA-rtPA NBs. (A) S1PR1 immunofluorescence staining results of excised rat mesenteric arterioles thrombosis and colocalization with DiI-labeled NBs (scale bar: 100 μm). (B) Quantitative fluorescence results of DiI in the rat mesenteric arterioles thrombosis regions. (C) S1PR1 immunofluorescence staining results of excised rat inferior vena cava thrombosis and colocalization with DiI-labeled NBs (scale bar: 100 μm). (D) Quantitative fluorescence results of DiI in the rat inferior vena cava thrombosis regions. Error bars: mean ± SD (*n* = 3). The statistical significance is indicated by ^*^ *P* < 0.05, ^**^ *P* < 0.01, ^***^ *P* < 0.001, determined using one-way ANOVA.

At last, the mechanism of the rapid and excellent targeting effect of S1P@CD-PLGA-rtPA NBs on thrombus was investigated at the pathological section level. When ferric chloride is applied to blood vessels, the chemical oxidation of Fe leads to vascular endothelial injury, causing platelet activation and adhesion and thrombus forming [32]. The S1PR1 immunofluorescence staining and colocalization with DiI-labeled NBs of rat mesenteric arterioles thrombosis sections (Fig. 4A) and rat inferior vena cava thrombosis sections (Fig. 4C) showed that there was a good fluorescence colocalization between DiI-labeled S1P@CD-PLGA-rtPA NBs and highly expressed S1PR1 in the thrombosis regions, indicating that the specific S1P–S1PR1 interaction contributes to the rapid and stable targeting of S1P@CD-PLGA-rtPA NBs to the thrombosis regions. Moreover, the quantitative fluorescence results of DiI in rat mesenteric arterioles thrombosis regions (Fig. 4B) as well as in rat inferior vena cava thrombosis regions (Fig. 4D) confirmed that the DiI fluorescence intensity of S1P@CD-PLGA-rtPA NBs on thrombosis regions was significantly higher than that of saline and CD-PLGA-rtPA NBs, demonstrating that S1P@CD-PLGA-rtPA NBs has an excellent thrombosis targeting effect due to the specific S1P–S1PR1 interaction.

### In vivo distribution and safety evaluation of S1P@CD-PLGA-rtPA NBs

**Fig. 5.**
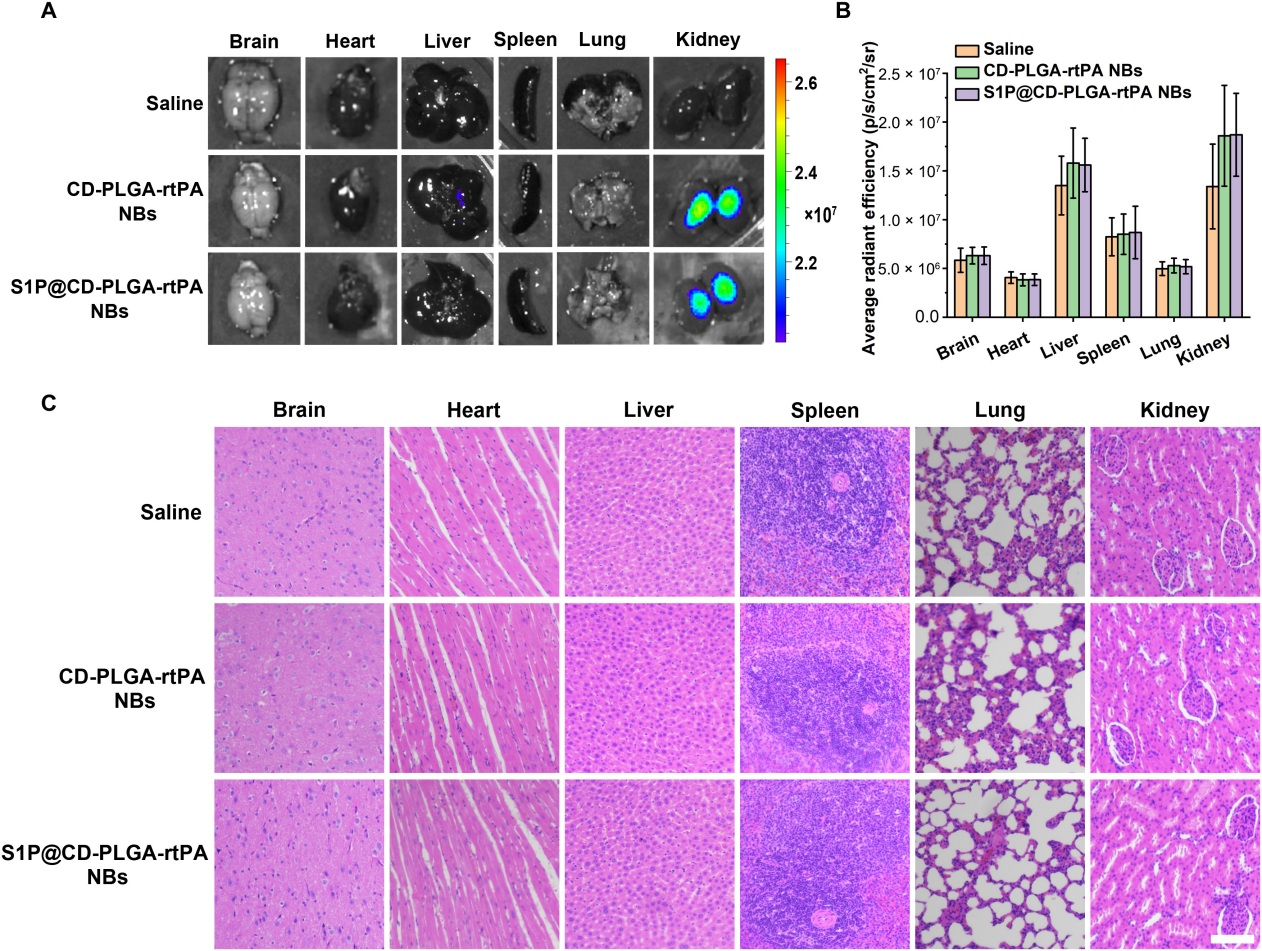
In vivo distribution and safety evaluation of S1P@CD-PLGA-rtPA NBs in rat. (A) Ex vivo near-infrared fluorescence imaging of excised major organs (brain, heart, liver, spleen, lung, and kidney) after intravenous injection of saline, CD-PLGA-rtPA NBs, and S1P@CD-PLGA-rtPA NBs for 24 h, respectively. (B) Fluorescence quantitative results and (C) H&E staining images of excised major organs (brain, heart, liver, spleen, lung, and kidney) (scale bar: 100 μm). Error bars: mean ± SD (*n* = 3).

The in vivo distribution and biosafety of S1P@CD-PLGA-rtPA NBs were evaluated in healthy rats. The near-infrared fluorescence results of the main organs after intravenous injection of saline, DiR-labeled CD-PLGA-rtPA NBs and DiR-labeled S1P@CD-PLGA-rtPA NBs for 24 h showed that both CD-PLGA-rtPA NBs and S1P@CD-PLGA-rtPA NBs were mainly distributed in the kidney and liver (Fig. 5A and B), which was mainly determined by the size, zeta potential, and surface modification of NBs [33]. Besides, as shown in Fig. 5C, the hematoxylin and eosin (H&E) stainning results of the main organs sections demonstrated that the histomorphology of each organ section injected with S1P@CD-PLGA-rtPA NBs was clear without obvious histopathological abnormalities or lesions, indicating that S1P@CD-PLGA-rtPA NBs have an excellent biosafety in vivo application.

### In vivo targeted thrombolysis by S1P@CD-PLGA-rtPA NBs combined with ultrasound in rat models

On the basis of the excellent targeting effect of S1P@CD-PLGA-rtPA NBs in rat mesenteric arterioles thrombosis and rat inferior vena cava thrombosis, in vivo targeted thrombolysis by S1P@CD-PLGA-rtPA NBs combined with ultrasound in ferric-chloride-induced rat mesenteric arterioles thrombosis and rat inferior vena cava thrombosis was further performed.

**Fig. 6.**
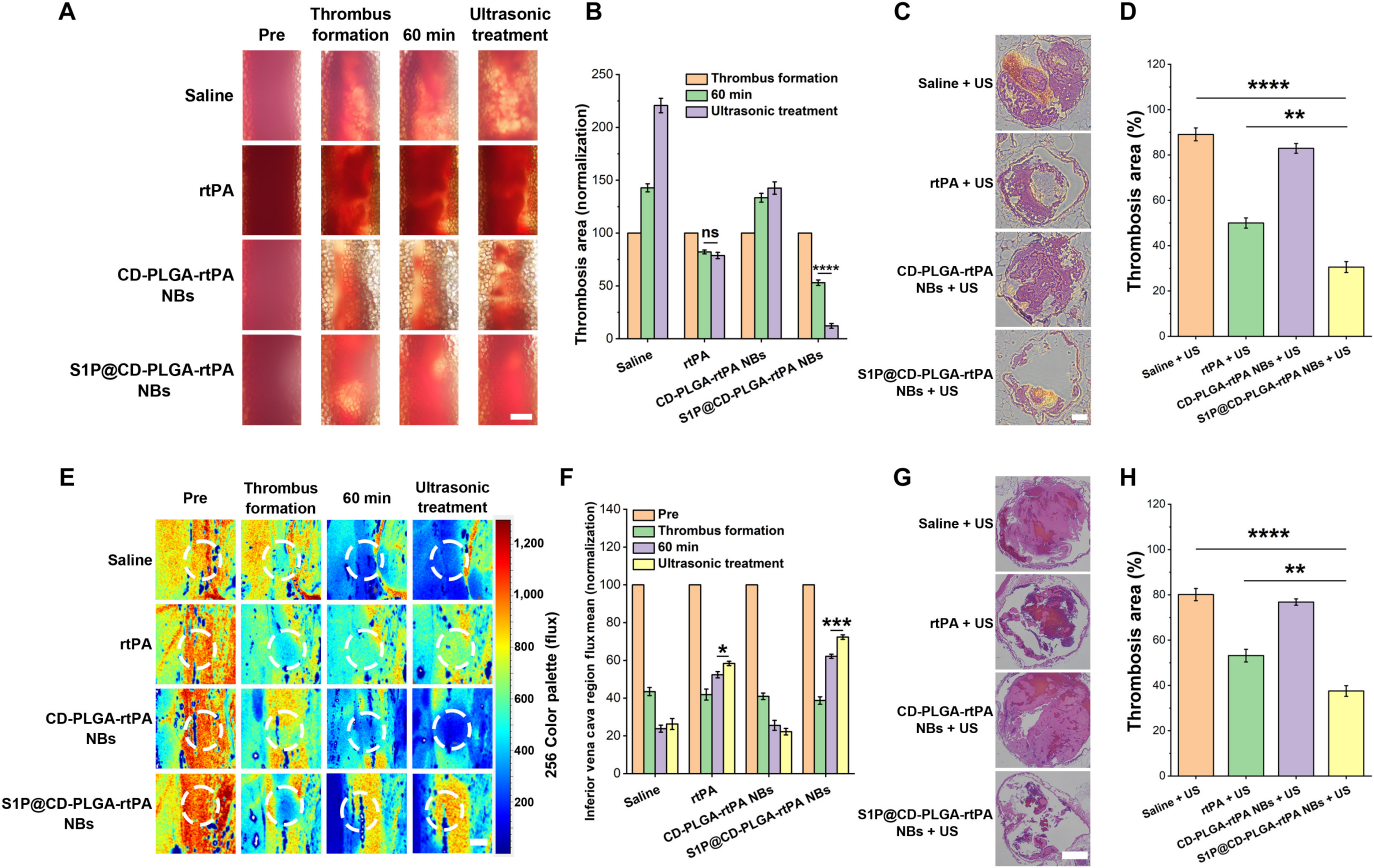
In vivo targeted thrombolysis by S1P@CD-PLGA-rtPA NBs combined with ultrasound in rat models. (A) Microscopic images of ferric-chloride-induced rat mesenteric arterioles thrombosis formation and thrombolysis by intravenous injection of saline, rtPA, CD-PLGA-rtPA NBs, and S1P@CD-PLGA-rtPA NBs, respectively (scale bar: 200 μm). (B) Normalized quantitative results of thrombosis area in optical microscopy. (C) H&E staining images of excised rat mesenteric arterioles thrombosis (scale bar: 50 μm) and (D) quantitative results of the thrombosis proportion. (E) Laser speckle blood flow images of ferric-chloride-induced rat inferior vena cava thrombosis formation and thrombolysis by intravenous injection of saline, rtPA, CD-PLGA-rtPA NBs, and S1P@CD-PLGA-rtPA NBs, respectively (scale bar: 1 mm, the white frame represents the area of thrombus). (F) Normalized quantitative analysis of blood flow of rat inferior vena cava. (G) H&E staining images of excised rat inferior vena cava thrombosis (scale bar: 500 μm) and (H) quantitative results of the thrombosis proportion. Error bars: mean ± SD (*n* = 5). The statistical significance is indicated by ^*^ *P* < 0.05, ^**^ *P* < 0.01, ^***^ *P* < 0.001 and ^****^ *P* < 0.0001, determined using one-way ANOVA.

First, the results of optical microscopy in Fig. 6A intuitively displayed the formation of thrombus in rat mesenteric arterioles, and the change of thrombus clots after injection of different samples (saline, rtPA, CD-PLGA-rtPA NBs, and S1P@CD-PLGA-rtPA NBs), respectively. The normalized quantitative results of thrombosis area in optical microscopy are shown in Fig. 6B. The optical microscopy results indicated that white clots appeared in the blood vessels when thrombus formation. After intravenous injection of different samples for 60 min, the normalized quantitative results of thrombosis area were 147.78 ± 3.83% (saline injection), 82.11 ± 1.85% (rtPA injection), 133.42 ± 4.17% (CD-PLGA-rtPA NBs injection), and 53.03 ± 2.57% (S1P@CD-PLGA-rtPA NBs injection), respectively. Results indicated that injection of S1P@CD-PLGA-rtPA NBs had the best thrombolysis effect because they specifically targeted to the thrombosis lesions and released rtPA for thrombolysis. Considering of the acoustic response properties of NBs, the external ultrasound was applied to effectively promote the release of rtPA in S1P@CD-PLGA-rtPA NBs to further enhance the thrombolysis effect. After the exposure of external ultrasound, the normalized thrombosis area for S1P@CD-PLGA-rtPA NBs injection group significantly reduced to 12.16 ± 2.25%, and the *P* value was 0.00006 compared with no external ultrasound applied (53.03 ± 2.57%). The reason may be that: (a) NBs vibrated under the excitation of external ultrasound, which benefits for promoting the effective release of the loaded rtPA. (b) NBs could induce ultrasonic cavitation effect and radiation force under ultrasound irradiation, and the resulting mechanical force and thermal effect could lead to the fragmentation and dissolution of thrombus clots [34]. (c) The microjet induced by the ultrasonic cavitation effect also enhanced the thrombus penetration depth of rtPA, which further improved the thrombolytic effect [35].

Furthermore, the H&E staining images of excised rat mesenteric arterioles thrombosis (Fig. 6C) and quantitative results of the thrombosis proportion (Fig. 6D) showed that the thrombosis area were 89.08 ± 2.84% (saline + US), 49.98 ± 2.25% (rtPA + US), 82.91 ± 2.14% (CD-PLGA-rtPA NBs + US), and 30.54 ± 2.40% (S1P@CD-PLGA-rtPA NBs + US), respectively, demonstrating that the targeted thrombolysis strategy of S1P@CD-PLGA-rtPA NBs combined with the external ultrasound (S1P@CD-PLGA-rtPA NBs + US) had the optimal in vivo thrombolysis effect, which has 2.92-fold reduction of thrombosis area to that of “saline + US” (*P* = 0.00002) as well as 1.64-fold reduction of thrombosis area to that of “rtPA + US” (*P* = 0.001).

In addition, the targeted thrombolysis experiment of S1P@CD-PLGA-rtPA NBs in rat inferior vena cava thrombosis with the addition of external ultrasound was also studied. The laser speckle blood flow imaging in Fig. 6E showed the blood flow of rat inferior vena cava when the thrombosis formed and changed after the injection of different samples (saline, rtPA, CD-PLGA-rtPA NBs, and S1P@CD-PLGA-rtPA NBs), respectively. The normalized quantitative results of blood flow of rat inferior vena cava were displayed in Fig. 6F. First, it was found that there was a notable reduction in blood flow at the thrombosis lesions. After intravenous injection of different samples for 60 min, the normalized blood flow quantitative results of thrombosis area were 23.83 ± 1.87% (saline injection), 52.37 ± 1.64% (rtPA injection), 25.56 ± 2.62% (CD-PLGA-rtPA NBs injection), and 62.17 ± 1.10% (S1P@CD-PLGA-rtPA NBs injection), respectively. Thus, the results indicated that injection of S1P@CD-PLGA-rtPA NBs had the best thrombolysis effect because they specifically targeted to the thrombosis lesions and released rtPA for thrombolysis. Moreover, the external ultrasound can be applied to effectively promote the release of rtPA in S1P@CD-PLGA-rtPA NBs to further enhance the thrombolysis effect (72.33 ± 1.23% of normalized blood flow), which has 1.16-fold enhancement of normalized blood flow compared to that of no external ultrasound applied (*P* = 0.0009). Besides, the H&E staining images of excised rat inferior vena cava thrombosis (Fig. 6G) and quantitative results of the thrombus proportion (Fig. 6H) showed that the thrombosis area were 80.13 ± 2.69% (saline + US), 53.18 ± 2.79% (rtPA + US), 76.82 ± 1.39% (CD-PLGA-rtPA NBs + US), and 37.54 ± 2.35% (S1P@CD-PLGA-rtPA NBs + US), respectively, confirming that the targeted thrombolysis strategy of S1P@CD-PLGA-rtPA NBs combined with the external ultrasound had the optimal in vivo thrombolysis effect, which has 2.13-fold reduction of thrombosis area to that of “saline + US” (*P* = 0.00007) as well as 1.42-fold reduction of thrombosis area to that of “rtPA + US” (*P* = 0.004).

## Conclusion

In this study, novel S1P@CD-PLGA-rtPA NBs with diameters of approximately 220 nm, stable properties, good biosafety, and excellent ultrasound enhancement effect were designed and constructed. The in vitro drug release experiments and in vitro thrombolysis experiments proved that S1P@CD-PLGA-rtPA NBs could utilize their own acoustic response properties to achieve the efficient release of rtPA under the external ultrasound. Besides, animal experiment results of rat mesenteric arterioles thrombosis and rat inferior vena cava thrombosis confirmed that S1P@CD-PLGA-rtPA NBs had rapid and excellent thrombosis targeting imaging performance based on the specific interaction of S1P–S1PR1, which is highly expressed in the thrombosis regions. Furthermore, S1P@CD-PLGA-rtPA NBs that specifically targeting to the thrombosis regions could also respond to external ultrasound to achieve accurate and efficient delivery of rtPA to further enhance the thrombolysis effectiveness and efficiency. Overall, this study proposes a new idea and strategy of targeting thrombus in rats via the specific S1P–S1PR1 interaction. On this basis, the acoustic response properties of bubble carriers could be fully utilized by combining thrombus-targeted imaging and ultrasound-mediated drug delivery to construct a novel multifunctional nanocarrier for thrombus-targeted theranostic, which is expected to be applied in targeted diagnosis and treatment of thrombotic diseases in the future.

## Materials and Methods

### Materials

PLGA (acid terminated, lactide: glycolide = 85: 15, molecular weight: 23,000 to 37,000) was purchased from Jinan Daigang Biomaterial Co., Ltd. (China). Poly-vinyl alcohol (PVA, molecular weight: 31,000) was obtained from Sigma-Aldrich (USA). 3A-amino-3A-deoxy-(2AS,3AS)-β-cyclodextrin hydrate was purchased from TCI Development Co., Ltd. (China). N-(3-Dimethylaminopropyl)-N’-ethylcarbodiimide hydrochloride (EDC·HCl) and N-hydroxysulfosuccinimide sodium salt (Sulfo-NHS) were obtained from Aladdin Reagent Co., Ltd. (China). S1P (d18: 1) was purchased from Avanti Polar Lipids, Inc. (Catalog No. 860492, USA); rtPA (Actilyse) was obtained from Boehringer Ingelheim (Ingelheim am Rhein, Germany). Sulfur hexafluoride (SF_6_) with a purity of 99.99% was purchased from Anhui Qiangyuan Gas Co., Ltd. (China). Thrombin was purchased from Shanghai Yuanye Bio-Technology Co., Ltd. (China). Anti-S1P1/EDG1 antibody (ab11424), and Goat Anti-Rabbit IgG H&L (Alexa Fluor 647) (ab150079) were purchased from Abcam (USA). Dulbecco’s modified Eagle’s medium and FBS were obtained from Gibco (USA). DiI, cell counting kit-8 (CCK-8), and Bradford protein assay kit were purchased from Beyotime Biotechnology (China). DiR was purchased from Jiangsu KeyGEN Biotechnology Co., Ltd. (China).

### Synthesis of S1P@CD-PLGA-rtPA NBs

S1P@CD-PLGA-rtPA NBs were fabricated in 4 steps: (a) fabrication of PLGA-rtPA nanocapsules; (b) synthesis of CD-PLGA-rtPA nanocapsules by coupling with β-CD on the surface of PLGA-rtPA nanocapsules; (c) construction of S1P@CD-PLGA-rtPA nanocapsules by inclusion of S1P with CD cavity; (d) preparation of S1P@CD-PLGA-rtPA NBs by freeze-drying and SF_6_ gas-filling.

PLGA-rtPA nanocapsules were first fabricated by W/O/W double emulsion-solvent evaporation method as previously described [36]. Briefly, the PLGA powder (200 mg) was dissolved in methylene chloride (2 ml). Then, rtPA aqueous solution (0.4 ml, 1 mg/ml) and Span 80 were added to the organic solution and the emulsion was sonicated for 60 s (400 W) in an ice bath. After emulsification, a 5% (w/v) aqueous solution of PVA (5 ml) and Tween 80 was added and sonicated in the same way. Then, the double emulsion was poured into a 0.5% (w/v) PVA (20 ml) to homogeneously stir for 5 h at 25 °C. The final solution was separated by using the differential centrifugation methods and washed 3 times with Milli-Q water. For preparation of the fluorescently labeled PLGA-rtPA nanocapsules, the fluorescent dye DiI or DiR (0.2 ml, at a concentration of 1 mg/ml) was added into the dichloromethane solution when dissolving the PLGA powder, and then it was mixed evenly. The other preparation procedures were the same as described above.

Second, amino-β-CD was coupled to the surface of the carboxyl modified PLGA-rtPA nanocapsules by the EDC/NHS chemical coupling method. The prepared PLGA-rtPA nanocapsules were precipitated and resuspended in 4-holineethanesulfonic acid (MES) buffer (pH = 5.4). Next, an EDC solution (0.2 ml, 20 mg/ml) and sulfo-NHS solution (0.2 ml, 20 mg/ml) were added, and the carboxyl group on the surface of PLGA-rtPA nanocapsules was activated by shaking at 240 rpm for 30 min at 25 °C. Then, the solution was centrifuged at 7,800 rpm for 30 min. When the precipitate was collected and resuspended in PBS (pH = 7.4), an amino-β-CD aqueous solution (0.4 ml, 20 mg/ml) was added to the solution. The coupling reaction was performed by shaking overnight at 240 rpm with a constant temperature mixer at 25 °C. After the reaction, the sample was centrifuged at 7,800 rpm for 30 min, and the precipitate was collected and suspended in Milli-Q water.

Third, S1P was encapsulated in the CD cavity by the ultrasonic inclusion method. The S1P solution (50 μl, 0.1 μg/μl) was slowly added to the resuspended samples mentioned above and mixed evenly. Then, sample was ultrasonicated in a water bath for 90 min (25 to 35 °C) using an ultrasonic cleaner (240 W). After sonication, the sample was centrifuged at 7,800 rpm for 30 min, and the precipitate was collected and suspended in Milli-Q water.

Finally, aliquots of the nanosphere suspensions (2 ml) were transferred to penicillin bottles (5 ml) to be stored in vials, and lyophilized using a freeze dryer (VirTis Advantage, SP scientific, USA) with mannitol as a protective agent. After the drying cycle was completed, SF_6_ was introduced into the vials, which were sealed with a paraffin film for experimental analysis.

### Characterization of S1P@CD-PLGA-rtPA NBs

The size distribution and zeta potential of the S1P@CD-PLGA-rtPA NBs were measured using a Zetasizer (Nano ZS90, Malvern Instruments, UK) at a 90° scattering angle. Each sample was measured in triplicate. A SEM (Ultra Plus, Zeiss, Germany) and a TEM (JEM-2100, JEOL, Japan) were used to characterize the morphology of NBs. A high-resolution TEM with an energy-dispersive spectrometer (EDS) (Talos F200X, Thermo Fisher Scientific, USA) was used to characterize the elemental mapping and elemental content. Fourier transform infrared spectrometer (IRAffinity-1S, SHIMADZU, Japan) was used to characterize the chemical bonds of samples (scan times: 40, scan range: 400 to 4,000 cm^−1^, resolution: 2 cm^−1^) to verify the successful loading of rtPA. To characterize the in vitro stability of S1P@CD-PLGA-rtPA NBs, the hydrodynamic size, zeta potential, and PDI of the samples stored in PBS (pH = 7.4) and 10% FBS for 7 d at 25 °C were investigated using dynamic light scattering measurement.

### Ultrasound imaging enhancement of S1P@CD-PLGA-rtPA NBs

A phantom (composed of 3% agar, 86% distilled degassed water, and 11% glycerol) with 2-ml sample loading well was used to characterize the in vitro ultrasound imaging capabilities of samples. The ultrasound imaging was performed on a high-resolution ultrasound imaging system (Vevo 2100, VisualSonics, Canada) with the transducer (MS-250) at 21-MHz central frequency using both B-mode and contrast mode. The imaging settings for the system were center frequency of 21 MHz, intensity power of 30%, contrast gain of 30 dB, 2-dimensional gain of 18 dB, and dynamic range of 35 dB. Ultrasound images of water (as blank control) and S1P@CD-PLGA-rtPA NBs were acquired at predetermined time intervals (0, 3, 6, 12, 18, 24, and 30 min), and a grayscale mapping function was used to calibrate the ultrasonic echo intensity.

### Loading of rtPA in S1P@CD-PLGA-rtPA NBs

To measure the encapsulation efficiency and loading content of rtPA in S1P@CD-PLGA-rtPA NBs, a modified Bradford method with Bradford Protein Assay Kit (Beyotime Biotechnology, China) was used. First, a standard curve was established for quantitative determination of rtPA concentration. Then, 5 mg of S1P@CD-PLGA-rtPA NBs lyophilized powder was weighed and added into a 1.5-ml Eppendorf (EP) tube. Then, 0.5 ml of methylene chloride was added to fully dissolve the lyophilized powder. Next, 0.5 ml Milli-Q water was added and the samples were shaken at 360 rpm for 30 min using a constant temperature (37 °C) shaker. Finally, the supernatants were collected by centrifugation at 7,200 rpm for 5 min, and the content of rtPA in the samples was determined and calculated according to the standard curve.

The encapsulation efficiency and loading content of rtPA in S1P@CD-PLGA-rtPA NBs can be calculated according to Eqs. 1 and 2, respectively.

$$\begin{array}{c} \text{Encapsulation efficiency}\;\left(\mathrm{EE}\%\right)\\ =\frac{\text{weight of loading rtPA}}{\text{weight of dosing rtPA}}\times 100\% \end{array}$$ (1)

$$\begin{array}{c} \text{Drug loading content}\;\left(\mathrm{DLC}\%\right)\\ =\frac{\text{weight of loading rtPA}}{\text{weight of}\;S1P@\mathrm{CD}-\text{PLGA}-\text{rtPA}\;\mathrm{NBs}}\times 100\% \end{array}$$ (2)

### Cell culture

The rat carotid artery endothelial cells were purchased from Shanghai Fusheng Industrial Co., Ltd. (Shanghai, China). Cells were cultured in Dulbecco’s modified Eagle’s medium supplemented with 10% FBS and 100 μg/ml penicillin/streptomycin. Cells were maintained in a humidified cell culture incubator at 37 °C with 5% carbon dioxide (CO_2_). The culture medium was refreshed every 2 to 3 d. When the cells reached 70 to 80% confluence, they were digested with 0.25% trypsin–EDTA solution, harvested by centrifugation, and resuspended in PBS for experimental use.

### Cell viability assay

The cytotoxicity of S1P@CD-PLGA-rtPA NBs in rat carotid artery endothelial cells was determined via CCK-8 cell proliferation assay based on a modified manufacturer’s protocol. Briefly, rat carotid artery endothelial cell was seeded with 96-well plates at a density of 1 × 10^4^ cells per well. After 24 h of culture, different concentrations of S1P@CD-PLGA-rtPA NBs (10% [v/v], NBs at a concentration of 0.2, 1, and 5 mg/ml, respectively) were added into the cell plate. After 1, 2, 4, 8, 12, 24, and 48 h incubation, the CCK-8 solution was added to each well and incubated for another 1 h. The absorbance of each well was measured at 450/650 nm by using a microplate reader (Multiskan Sky, Thermo Fisher Scientific, USA).

### Erythrocyte hemolysis assay

Blood was collected from SD rats and centrifuged at 2,500 × g for 10 min, the supernatant was removed, and the erythrocytes was obtained. The precipitate was washed and resuspended with saline and then centrifuged again with the same parameters 3 times. Then, the treated erythrocytes were made into a 1% suspension with saline for experimental use. After dividing the erythrocyte suspension equally into centrifuge tubes (1 ml/tube), 1 ml of different samples were added and incubated at 25 °C for 4 h (saline was used as a negative control group without hemolytic effect, water was used as a positive control group with hemolytic effect, and S1P@CD-PLGA-rtPA NBs at concentrations of 0.2, 1, and 5 mg/ml were used). The incubation solution was centrifuged at 10,000 × g for 1 min, and the supernatant was added into a 96-well plate. The absorbance (optical density) at 541 nm was measured using a microplate reader (Multiskan Sky, Thermo Fisher Scientific, USA) and Eq. 3 was used to calculate the hemolysis rate (HR) of the samples.

$$\text{HR}=\frac{\left({\mathrm{OD}}_{\text{Sample}}-{\mathrm{OD}}_{\text{Saline}}\right)}{\left({\mathrm{OD}}_{\text{Water}}-{\mathrm{OD}}_{\text{Saline}}\right)}\times 100\%$$ (3)

### Platelet aggregation assay

Aggregation of platelets in the presence of S1P@CD-PLGA-rtPA NBs was assessed using a spectrophotometric method. Blood was collected from healthy SD rats and then fresh platelets were isolated and prepared into platelet suspension at a concentration of 1 × 10^9^/ml using saline. 0.5 ml of platelet suspension was added to a 24-well plate followed by addition of saline (negative control), thrombin (positive control, 25 IU), and S1P@CD-PLGA-rtPA NBs (0.5 ml, at a concentration of 0.2, 1, and 5 mg/ml). Aggregation of platelets was assessed at 650 nm every 300 s (Multiskan Sky, Thermo Fisher Scientific, USA). Platelet aggregation was demonstrated by the decrease in absorbance due to reduced turbidity. Each sample was assayed in triplicate (*n* = 3).

### In vitro release of rtPA

To measure in vitro release of rtPA from S1P@CD-PLGA-rtPA NBs, first, 5 mg of S1P@CD-PLGA-rtPA NBs lyophilized powder was weighed and added into a 1.5-ml EP tube. Then, 1 ml of Milli-Q water was added to fully dissolve the lyophilized powder. Next, samples were shaken by a constant temperature shaker (37 °C, 200 rpm). After shaking for a certain time, the EP tube was centrifuged at 7,200 rpm for 5 min, and 0.1 ml of supernatant was taken out to determine the rtPA content by the Bradford method described above. Subsequently, 0.1 ml of Milli-Q water was added into the EP tube, and samples were continuously shaken by a constant temperature shaker (37 °C, 200 rpm). The continuous release of rtPA in the S1P@CD-PLGA-rtPA NBs was determined, and the drug release curve was plotted.

Furthermore, to measure in vitro release of rtPA from S1P@CD-PLGA-rtPA NBs under the external ultrasound, similarly, 5 mg of S1P@CD-PLGA-rtPA NBs lyophilized powder was weighed and added into a penicillin bottle (3 ml) and 1 ml of Milli-Q water was added to fully dissolve the lyophilized powder. Then, a variety of external ultrasonic strategies (US 1: frequency of 1 MHz, intensity of 0.25 W/cm^2^, ultrasonic for 20 min; US 2: frequency of 1 MHz, intensity of 1.00 W/cm^2^, ultrasonic for 20 min; US 3: frequency of 1 MHz, intensity of 2.50 W/cm^2^, ultrasonic for 20 min; US 4: frequency of 1 MHz, intensity of 2.50 W/cm^2^, ultrasonic for 5 min and then pause for 1 min as a cycle, repetition for 6 times; US 5: frequency of 1 MHz, intensity of 2.50 W/cm^2^, ultrasonic for 10 min and then pause for 2.5 min as a cycle, repetition for 3 times; US 6: frequency of 1 MHz, intensity of 2.50 W/cm^2^, ultrasonic for 15 min, pause for 5 min and then ultrasonic for another 15 min) were applied to the samples using an ultrasound therapeutic instrument (DM-200B, Demai Technology Co., Ltd., China). Specifically, the external ultrasound was applied using the treatment transducer with an ultrasound radiation area of 2 cm^2^ attached to the bottom of the penicillin bottle coated with coupling agent. After ultrasonic application, the samples were centrifuged at 7,200 rpm for 5 min, and 0.1 ml of supernatants was collected to determine the rtPA content by the Bradford method described above.

### In vitro thrombolysis experiments

Blood samples were collected from SD rats and bathed in water at 37 °C for 2 h until thrombus clot was formed. Then, the thrombus clot was cut into a uniform mass (clot 1) and put into tubes containing 2 ml of saline. Next, the tubes were added with 1 ml of different samples (saline, rtPA, CD-PLGA-rtPA NBs, S1P@CD-PLGA-rtPA NBs, and S1P@CD-PLGA-rtPA NBs + US, *n* = 3) and gently shaken on a constant temperature shaker at 37 °C. Two hours later, the thrombus clot was taken out from the tubes, dried, and weighed (clot 2). (Notes: As for the group of “S1P@CD-PLGA-rtPA NBs + US”, when S1P@CD-PLGA-rtPA NBs were added into the tube for 1 h, external ultrasound was applied using an ultrasonic therapeutic instrument [DM-200B, Demai Technology Co., Ltd., China]. External ultrasonic parameters: frequency of 1 MHz, intensity of 2.50 W/cm^2^, ultrasonic for 15 min, pause for 5 min, and then ultrasonic for 15 min)

The thrombolysis rate was calculated according to Eq. 4.

$$\begin{array}{c} \text{Thrombolysis rate}\\ =\frac{\text{weight of clot}\;1-\text{weight of clot}\;2}{\text{weight of clot}\;1}\times 100\% \end{array}$$ (4)

### In vivo S1P@CD-PLGA-rtPA NBs thrombosis targeting effect in rat mesenteric arterioles thrombosis

All animal experiments were performed according to the Guidelines for Care and Use of Laboratory Animals established by Medical School of Southeast University Institutional Animal Care and Use Committee (20210302039). For the animal experiment of S1P@CD-PLGA-rtPA NBs targeting rat mesenteric arterioles thrombosis, first, SD rats (male, 220- to 250-g body weight) were anesthetized by subcutaneous injection of the urethane (25%, 5 μl/g body weight). An incision was performed through the abdominal wall to expose the mesenteric arteriole, and a piece of filter paper saturated with the FeCl_3_ (10% [w/v]) solution was applied topically for 2 min [37]. After the filter paper was removed, the formation and development of thrombus in real time was monitored by an inverted fluorescent microscope (ECLIPSE Ti2-U, Nikon, Japan). When the thrombosis occurred after 10 min, 800 μl of saline, DiI-labeled CD-PLGA-rtPA NBs (NBs at a concentration of 5.0 mg/ml, *n* = 3), and DiI-labeled S1P@CD-PLGA-rtPA NBs (NBs at a concentration of 5.0 mg/ml, *n* = 3) were injected into the tail vein, respectively. The inverted fluorescence microscope was used to characterize the targeting effect of NBs on the thrombus site of mesenteric arterioles within 60 min. Finally, the rats were sacrificed and the mesenteric arterioles were excised, fixed with 4% paraformaldehyde fixative overnight at 4 °C in darkness for subsequent pathological section analysis by S1PR1 immunofluorescence staining. The mean fluorescence intensity was calculated using the ImageJ software (NIH, USA).

### In vivo S1P@CD-PLGA-rtPA NBs thrombosis targeting effect in rat inferior vena cava thrombosis

For the animal experiment of S1P@CD-PLGA-rtPA NBs targeting rat inferior vena cava thrombosis, SD rats (male, 220- to 250-g body weight) were intraperitoneally anesthetized with pentobarbital sodium solution (1%, 10 μl/g body weight). An incision was made through the abdominal wall to expose the inferior vena cava, and a piece of filter paper saturated with FeCl_3_ (10% [w/v]) solution was applied topically for 2 min [38]. After the filter paper was then removed, the formation and development of thrombus was monitored via an ultrasound imaging system (Vevo 2100, VisualSonics, Canada) with an MS-250 transducer (center frequency of 21 MHz, intensity power of 10%, and contrast gain of 25 dB). When the thrombosis occurred after 15 min, 800 μl of saline, DiR-labeled CD-PLGA-rtPA NBs (NBs at a concentration of 5.0 mg/ml, *n* = 3) and DiR-labeled S1P@CD-PLGA-rtPA NBs (NBs at a concentration of 5.0 mg/ml, *n* = 3) were injected into the tail vein, respectively, and the site of the inferior vena cava thrombosis was monitored by using an ultrasound imaging system (Vevo 2100, VisualSonics, Canada) with an MS-250 transducer within 60 min. Specifically, the MS-250 transducer was placed vertically above the inferior vena cava coated with coupling agent, and the depth of focus was 16 mm. Subsequently, the rats were sacrificed and the inferior vena cavas were excised for near-infrared fluorescence imaging (IVIS Spectrum, PerkinElmer, USA).

To verify the thrombosis targeting effect of S1P@CD-PLGA-rtPA NBs at the pathological section level, 800 μl of saline, DiI-labeled CD-PLGA-rtPA NBs (NBs at a concentration of 5.0 mg/ml, *n* = 3), DiI-labeled S1P@CD-PLGA-rtPA NBs (NBs at a concentration of 5.0 mg/ml, *n* = 3) were injected into the tail vein, respectively, when the rat inferior vena cava thrombosis formed. After 60 min, the rats were sacrificed and the inferior vena cavas were excised, fixed with 4% paraformaldehyde fixative overnight at 4 °C in darkness for subsequent pathological section analysis by S1PR1 immunofluorescence staining. The mean fluorescence intensity was calculated using the ImageJ software (NIH, USA).

### Targeted thrombolysis of rat mesenteric arterioles thrombosis

The rat mesenteric arterioles thrombosis model was performed according to the above-mentioned method. When the thrombus formed, 800 μl of saline, rtPA, CD-PLGA-rtPA NBs (NBs at a concentration of 5.0 mg/ml, *n* = 5), and S1P@CD-PLGA-rtPA NBs (NBs at a concentration of 5.0 mg/ml, *n* = 5) were injected into the tail vein, respectively, and the inverted fluorescence microscope was used to characterize the change of mesenteric vascular thrombus within 60 min. Subsequently, an ultrasonic therapeutic instrument (DM-200B, Demai Technology Co., Ltd., China) was used to apply external ultrasound to the thrombosis site of mesenteric arterioles for promoting efficient delivery of rtPA (external ultrasonic parameters: frequency of 1 MHz, intensity of 2.50 W/cm^2^, ultrasonic for 15 min, pause for 5 min, and then ultrasonic for 15 min). Finally, the rats were sacrificed and the mesenteric arterioles were excised, fixed with 4% paraformaldehyde fixative overnight at 4 °C in darkness for subsequent pathological section analysis by H&E staining. Moreover, quantification of the thrombosis area (% vascular lumen) by measuring the cross-sectional areas of the clots and the vessel in the histological images using the ImageJ software (NIH, USA) provided a rough estimate of the thrombosis area.

### Targeted thrombolysis of rat inferior vena cava thrombosis

The rat inferior vena cava thrombosis model was performed according to the abovementioned method. The blood flow of rat inferior vena cava was characterized by a laser speckle blood flow imaging system (FLPI-2 Pro, Moor Instruments, UK). When the thrombus formed, 800 μl of saline, rtPA, CD-PLGA-rtPA NBs (NBs at a concentration of 5.0 mg/ml, *n* = 5), and S1P@CD-PLGA-rtPA NBs (NBs at a concentration of 5.0 mg/ml, *n* = 5) were injected into the tail vein, respectively. Sixty minutes later, an ultrasonic therapeutic instrument (DM-200B, Demai Technology Co., Ltd., China) was used to apply external ultrasound to the thrombosis site of rat inferior vena cava for promoting efficient delivery of rtPA (external ultrasonic parameters: frequency of 1 MHz, intensity of 2.50 W/cm^2^, ultrasonic for 15 min, pause for 5 min, and then ultrasonic for 15 min). Finally, the rats were sacrificed and the inferior vena cavas were excised, fixed with 4% paraformaldehyde fixative overnight at 4 °C in darkness for subsequent pathological section analysis by H&E staining. Furthermore, quantification of the thrombosis area (% vascular lumen) by measuring the cross-sectional areas of the clots and the vessel in the histological images using the ImageJ software (NIH, USA) provided a rough estimate of the thrombosis area.

### In vivo distribution and safety evaluation

SD rats (male, 220- to 250-g body weight) were intraperitoneally anesthetized with pentobarbital sodium solution (1%, 10 μl/g body weight). Afterward, 800 μl of saline, DiR-labeled CD-PLGA-rtPA NBs (NBs at a concentration of 5.0 mg/ml, *n* = 3) and DiR-labeled S1P@CD-PLGA-rtPA NBs (NBs at a concentration of 5.0 mg/ml, *n* = 3) were injected into the tail vein, respectively. After intravenous injection for 24 h, the rats were sacrificed and the major organs (brain, heart, liver, spleen, lung, and kidney) were excised for near-infrared fluorescence imaging (IVIS Spectrum, PerkinElmer, USA) to characterize the in vivo distribution of the NBs. Lastly, the major organs (brain, heart, liver, spleen, lung, and kidney) were fixed with 4% paraformaldehyde fixative overnight at 4 °C in darkness for subsequent pathological section analysis by H&E staining.

### Statistical analysis

Statistical calculations were performed in GraphPad Prism Version 9.5, and quantitative data were presented as mean ± standard deviation (SD) from sample numbers (*n*). Statistical comparisons were made by unpaired 2-tailed Student *t* test (between 2 groups) and 1-way analysis of variance (ANOVA) with post hoc Tukey honestly significant difference tests (for multiple comparisons). ^*^P value < 0.05 was considered statistically significant; ^**^P < 0.01, ^***^P < 0.001, and ^****^P < 0.0001 were extremely significant; and ns was considered no significant difference. ImageJ software (National Institutes of Health, USA) was used for densitometric analysis of fluorescence intensity in confocal images.

## Data Availability

The data are available from the authors upon a reasonable request.
