# Supplementary material for: Controllable Thrombolysis Using a Nanobubble-Imaging-Guided rtPA Targeted Delivery Strategy
Source: BME Front. 2024 Mar 26;5:0040. doi: 10.34133/bmef.0040 (PMC10976949; doi:10.34133/bmef.0040)
Supplement: Supplementary 1 — Figs. S1 to S4 [file bmef.0040.f1.pdf]

FRONT MATTER

Controllable thrombolysis using a nanobubble imaging guided rtPA targeted delivery strategy

Jian Tang[1], Huiting Xu[1], Mingxi Li[1], Yang Liu[1], Fang Yang[1*]

[1] State Key Laboratory of Digital Medical Engineering, Jiangsu Key Laboratory for Biomaterials and Devices, School of Biological Sciences and Medical Engineering, Southeast University, Nanjing 210096, China

*Address correspondence to: Fang Yang, yangfang2080@seu.edu.cn

SUPPLEMENTARY MATERIALS

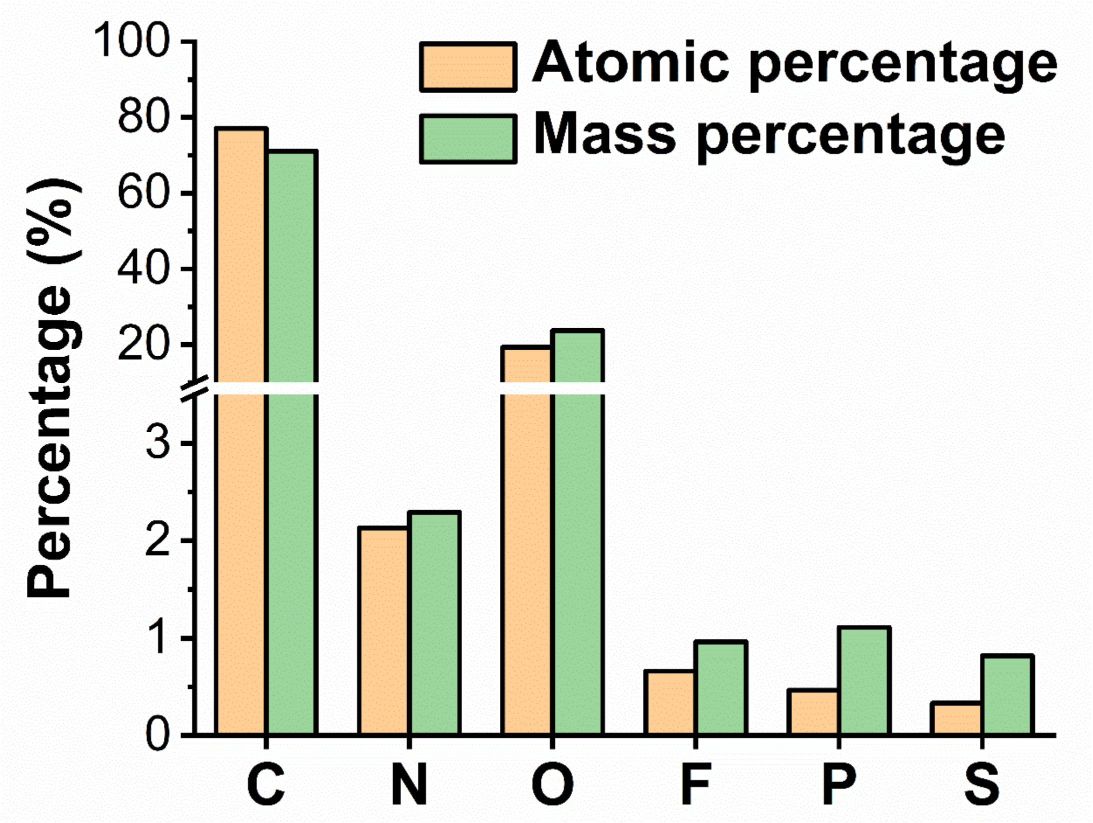

Fig. S1. The elemental contents of carbon (C), nitrogen (N), oxygen (O), fluorine (F), phosphorus (P), sulfur (S) elements in S1P@CD-PLGA-rtPA NBs.

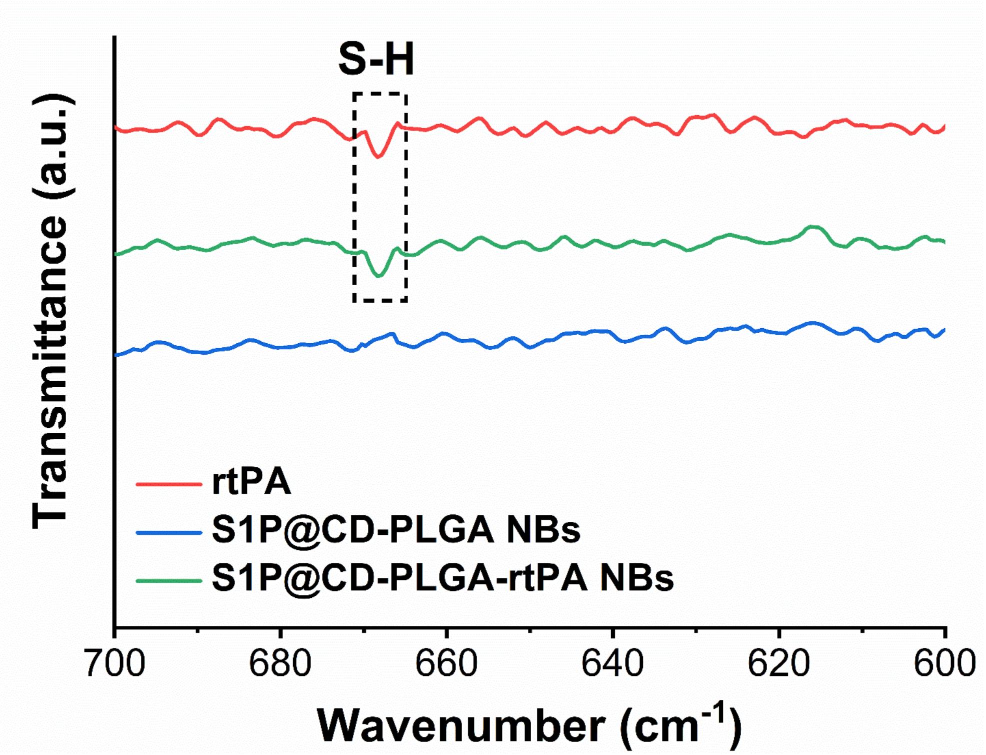

Fig. S2. Infrared absorption spectra of rtPA, S1P@CD-PLGA NBs and S1P@CD-PLGA-rtPA NBs from 700 cm^{-1} to 600 cm^{-1}.

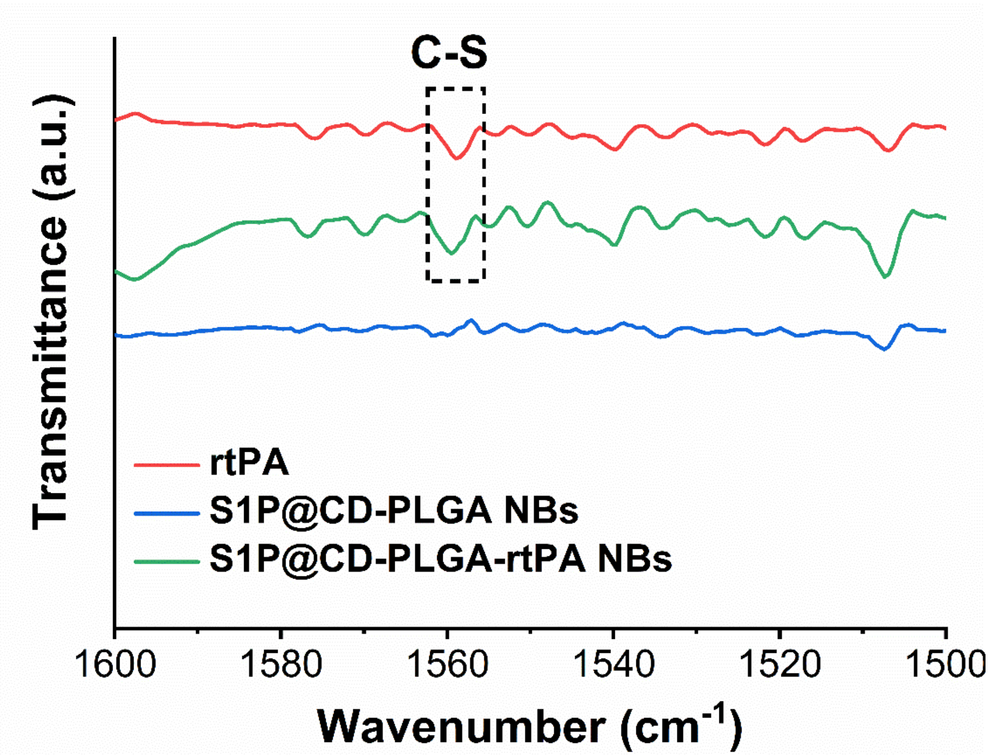

Fig. S3. Infrared absorption spectra of rtPA, S1P@CD-PLGA NBs and S1P@CD-PLGA-rtPA NBs from 1600 cm^{-1} to 1500 cm^{-1}.

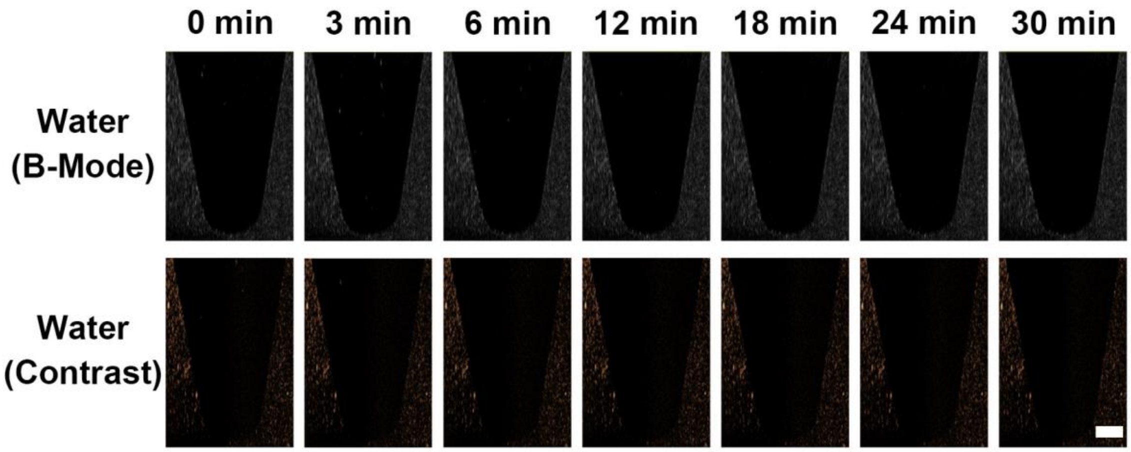

Fig. S4. B-Mode and Contrast mode ultrasound images of water at different time intervals (scale bar: 2 mm).
